# Supplementary material for: Design and Fabrication of Pneumatically Actuated Valveless Pumps
Source: Micromachines (Basel). 2021 Dec 23;13(1):16. doi: 10.3390/mi13010016 (PMC8780249; doi:10.3390/mi13010016)
Supplement: Supplementary file 1 [file micromachines-13-00016-s001.zip › supplementary document S1.pdf]

Supplementary document S1

The strain energy of bending (U_b) and stretching (U_S) of the membrane can be expressed as:

$$U_b = \frac{Eh^3}{24(1-\nu^2)} \int_0^{2\pi}\int_0^a \left[\left(\frac{\partial^2 w}{\partial r^2}\right)^2 + \frac{1}{r^2}\left(\frac{\partial w}{\partial r}\right)^2 + \frac{2\nu}{r}\frac{\partial w}{\partial r}\frac{\partial^2 w}{\partial r^2} \right] \cdot r \cdot dr \cdot d\theta \tag{1}$$

$$U_S = \frac{Eh^3}{24(1-\nu^2)} \int_0^a \left(\left[\frac{du}{dr} + \frac{1}{2}\left(\frac{dw}{dr}\right)^2 \right]^2 + \left(\frac{u}{r}\right)^2 + \frac{2\nu u}{r}\left(\frac{du}{dr} + \frac{1}{2}\left(\frac{dw}{dr}\right)^2 \right) \right) rdr \tag{2}$$

Most of circular diaphragms are considered to have clamped boundary conditions, that is

$$\frac{\partial w^*(0)}{\partial r^*} = 0; \quad w^*(1) = 0 \tag{3}$$

Here $w^*(r^*)=w/wo$, $r^*=r/a$. w_o is the characteristic displacement of $w(r)$. $w^*(r^*)$ is assumed to be expressed by

$$w^*(r^*) = \left(1 - r^{*2}\right)^2 \tag{4}$$

u also has to be satisfied by the boundary conditions, that is

$$u^*(0) = u^*(1) = 0 \tag{5}$$

Here $u^*(r^*)=u/u_o$, $r^*=r/a$. u_o is also the characteristic displacement of $u(r)$. So, the profile of u^* (r^*) can be assumed as:

$$u^*(r^*) = r^*\left(1 - r^*\right)^3 \tag{6}$$

The derivatives of w and u are acquired as:

$$\frac{\partial w}{\partial r} = \frac{w_o}{a}\frac{\partial w^*}{\partial r^*} = \frac{2w_o}{a}\left(1 - r^{*2}\right)\left(-2r^*\right) = \frac{-4w_o r^*}{a}\left(1 - r^{*2}\right) \tag{7}$$

$$\frac{\partial^2 w}{\partial r^2} = \frac{w_o}{a^2}\frac{\partial^2 w^*}{\partial r^{*2}} = \frac{-4w_o}{a^2}\left(1 - 3r^{*2}\right) \tag{8}$$

$$\frac{\partial u}{\partial r} = \frac{u_o}{a}\frac{\partial u^*}{\partial r^*} = \frac{u_o}{a}\left[\left(1 - r^{*2}\right)^3 + r^*(3)\left(1 - r^{*2}\right)^2\left(-2r^*\right) \right] = \frac{u_o}{a}\left(1 - r^{*2}\right)^2\left(1 - 7r^{*2}\right) \tag{9}$$

$$\frac{u}{r} = \frac{u_o}{a}\frac{u^*}{r^*} = \frac{u_o}{a}\left(1 - r^{*2}\right)^3 \tag{10}$$

Substituting Eqs (A-7) and (A-8) into normalization of Eq. (A-1), it could be obtained as

$$U_b = \frac{\pi Eh^3}{12(1-\nu^2)} \int_0^1 \left[\left(\frac{w_o}{a^2}\frac{\partial^2 w^*}{\partial r^{*2}}\right)^2 + \frac{1}{a^2 r^{*2}}\left(\frac{w_o}{a}\frac{\partial w}{\partial r}\right)^2 + \frac{2\nu}{ar^*}\frac{w_o \cdot \partial w^*}{a \cdot \partial r^*}\frac{w_o \cdot \partial^2 w^*}{a^2 \cdot \partial r^{*2}} \right] \cdot a^2 \cdot r^* \cdot dr^*$$

$$= \frac{\pi Eh^3 w_o^2}{12(1-\nu^2)a^2} \int_0^1 \left[\left(\frac{\partial^2 w^*}{\partial r^{*2}}\right)^2 + \frac{1}{r^{*2}}\left(\frac{\partial w}{\partial r}\right)^2 + \frac{2\nu}{r^*}\frac{\partial w^*}{\partial r^*}\frac{\partial^2 w^*}{\partial r^{*2}} \right] \cdot r^* \cdot dr^*$$

$$= \frac{4\pi Eh^3 w_o^2}{3(1-\nu^2)a^2} \int_0^1 \left[\left(1 - 3r^{*2}\right)^2 + \left(1 - r^{*2}\right)^2 + 2\nu\left(1 - r^{*2}\right)\left(1 - 3r^{*2}\right) \right] \cdot r^* \cdot dr^*$$

$$=\frac{4\pi Eh^3w_o^2}{3(1-\nu^2)a^2}\int_0^1\left[\left(1-6r^{*2}+9r^{*4}\right)+\left(1-2r^{*2}+r^{*4}\right)+2\nu\left(1-4r^{*2}+3r^{*4}\right)\right]\cdot r^*\cdot dr^*$$

$$=\frac{4\pi Eh^3w_o^2}{3(1-\nu^2)a^2}\left[\left(r^2-2r^{*4}+\frac{10}{6}r^{*6}\right)+\nu\left(r^{*2}-2r^{*4}+r^{*6}\right)\right]\Bigg|_0^1$$

$$=\frac{8\pi Eh^3}{9(1-\nu^2)}\cdot\frac{w_o^2}{a^2} \tag{11}$$

Similarly, Substituting Eqs (A-7), (A-9) and (A-10) into normalization of Eq. (A-2), it could be obtained as

$$U_S=\frac{\pi Eh}{(1-\nu^2)}\int_0^1\left[\left(\frac{u_o}{a}\frac{du^*}{dr^*}+\frac{w_o^2}{2a^2}\left(\frac{dw^*}{dr^*}\right)^2\right)^2+\left(\frac{u_o}{a}\frac{u^*}{r^*}\right)^2+2\nu\left(\frac{u_o}{a}\frac{du^*}{dr^*}+\frac{w_o^2}{2a^2}\left(\frac{dw^*}{dr^*}\right)^2\right)\cdot\left(\frac{u_o}{a}\frac{u^*}{r^*}\right)\right]\cdot a^2\cdot r^*\cdot dr^*$$

$$=\frac{\pi Eh}{(1-\nu^2)}\int_0^1\left[\left(\frac{u_o}{a}\left(1-r^{*2}\right)^2\left(1-7r^{*2}\right)+\frac{8w_o^2r^{*2}}{a^2}\left(1-r^{*2}\right)^2\right)^2+\left(\frac{u_o}{a}\left(1-r^{*2}\right)^3\right)^2+2\nu\left(\frac{u_o}{a}\left(1-r^{*2}\right)^2\left(1-7r^{*2}\right)\right.\right.$$
$$\left.\left.+\frac{8w_o^2r^{*2}}{a^2}\left(1-r^{*2}\right)^2\right)\left(\frac{u_o}{a}\left(1-r^{*2}\right)^3\right)\right]a^2\cdot r^*\cdot dr^*$$

$$=\frac{\pi Eh}{(1-\nu^2)}\int_0^1\left[\left(u_o^2\left(1-r^{*2}\right)^4\left(1-7r^{*2}\right)^2+\frac{16u_ow_o^2r^{*2}}{a}\left(1-r^{*2}\right)^4\left(1-7r^{*2}\right)+\frac{64w_o^4r^{*4}}{a^2}\left(1-r^{*2}\right)^4\right)+u_o^2\left(1-r^{*2}\right)^6\right.$$
$$\left.+2\nu\left(u_o^2\left(1-r^{*2}\right)^5\left(1-7r^{*2}\right)+\frac{8u_ow_o^2r^{*2}}{a}\left(1-r^{*2}\right)^5\right)\right]r^*dr^* \tag{12}$$

Integrating and simplifying Eq.(12), it could be obtained as:

$$U_S=\frac{\pi Eh}{(1-\nu^2)}\left\{\frac{u_o^2}{10}-\frac{4}{15}\frac{u_ow_o^2}{a}+\frac{32}{105}\frac{w_o^4}{a^2}+\frac{u_o^2}{14}+\frac{4\nu}{21}\frac{u_ow_o^2}{a^3}\right\}$$

$$=\frac{\pi Eh}{(1-\nu^2)}\left\{\frac{6u_o^2}{35}-\frac{4}{15}\frac{u_ow_o^2}{a}+\frac{32}{105}\frac{w_o^4}{a^2}+\frac{4\nu}{21}\frac{u_ow_o^2}{a^3}\right\} \tag{13}$$

During deforming, the radial displacement satisfies the principles of bending energy minimization in achieving equilibrium, i.e., $dUs/du_o = 0$, thus

$$\frac{dU_S}{du_o}=\frac{\pi Eh}{(1-\nu^2)}\left\{\frac{12u_o}{35}-\frac{4}{15}\frac{w_o^2}{a}+\frac{4\nu}{21}\frac{w_o^2}{a}\right\}=0 \tag{14}$$

Hence, the relation of u_o and w_o is obtained by:

$$u_o=\frac{(7-5\nu)}{9}\frac{w_o^2}{a} \tag{15}$$

Substituting Eq.(15) into Eq.(13) and then simplify as:

$$U_S=\frac{\pi Eh}{(1-\nu^2)}\left\{\frac{6}{35}\left(\frac{(7-5\nu)}{9}\frac{w_o^2}{a}\right)^2-\frac{4}{15}\left(\frac{(7-5\nu)}{9}\frac{w_o^2}{a}\right)\frac{w_o^2}{a}+\frac{32}{105}\frac{w_o^4}{a^2}+\frac{4\nu}{21}\left(\frac{(7-5\nu)}{9}\frac{w_o^2}{a}\right)\frac{w_o^2}{a}\right\}$$

$$=\frac{\pi Eh}{(1-\nu^2)}\left\{\frac{2}{35}\left(\frac{49-70\nu+25\nu^2}{27}\right)-\frac{4}{15}\left(\frac{(7-5\nu)}{9}\right)+\frac{32}{105}+\frac{4\nu}{21}\left(\frac{(7-5\nu)}{9}\right)\right\}\frac{w_o^4}{a^2}$$

$$=\frac{\pi Eh}{(1-\nu^2)}\left(\frac{190+140\nu-50\nu^2}{945}\right)\frac{w_o^4}{a^2}$$

$$= \frac{\pi Eh}{(1-\nu^2)} \frac{10(19-5\nu)(1+\nu)}{945} \frac{w_o^4}{a^2}$$

$$= \frac{2\pi Eh}{(1-\nu)} \frac{(19-5\nu)}{189} \frac{w_o^4}{a^2} \quad (16)$$

The external pneumatic energy due to pressure loading is integrated by

$$U_P = \int_0^{2\pi}\int_0^a Pwrdrd\theta = \frac{\pi P w_o a^2}{3} \quad (17)$$

For the deformation mechanism of the membrane, total energy (U_T) consisted of the strain energy, stretching energy, and external pneumatic energy. Therefore,

$$U_T = \frac{8\pi Eh^3}{9(1-\nu^2)} \frac{w_o^2}{a^2} + \frac{2\pi Eh}{189} \frac{(19-5\nu)}{(1-\nu)} \frac{w_o^4}{a^2} - \frac{\pi P w_o a^2}{3} \quad (18)$$

Therefore, the derivative of the total energy approaches to zero (i.e., $dU_T/dw_o=0$) as the membrane attains a maximum deformation. Thus, the value of wo/h can be expressed as

$$\frac{16}{3(1-\nu^2)}\left(\frac{w_o}{h}\right) + \frac{8(19-5\nu)}{63(1-\nu)}\left(\frac{w_o}{h}\right)^3 = \frac{Pa^4}{Eh^4} \quad (19)$$

Substituting the Poisson's ratio of 0.5 into equation (19), Equation (19) can be obtained as

$$\frac{64}{9}\left(\frac{w_o}{h}\right) + \frac{264}{63}\left(\frac{w_o}{h}\right)^3 = \frac{Pa^4}{Eh^4} \quad (20)$$

Equation (20) is used to predict the maximum deformation of membrane under the parameters such as the membrane's radius, thickness, Young's module, and applied air pressures.

Summary, the larger deflection of the membrane, i.e., $w_o/h \gg 1$, Eq. (20) can be simplified as follows:

$$\frac{w_o}{a} = 0.62\left(\frac{Pa}{Eh}\right)^{\frac{1}{3}} \quad (21)$$
